# Editorial: On the Planning, Control, and Perception of Soft Robotic End-Effectors

**DOI:** 10.3389/frobt.2021.795863

**Published:** 2021-11-22

**Authors:** Giuseppe Averta, Cosimo Della Santina, Fanny Ficuciello, Maximo A. Roa, Matteo Bianchi

**Affiliations:** ^1^ Department of Control and Computer Engineering, Politecnico di Torino, Turin, Italy; ^2^ Cognitive Robotics (CoR) Department, Delft University of Technology, Delft, Netherlands; ^3^ PRISMA Lab - DIETI (Dipartimento di Ingegneria Elettrica e delle Tecnologie dell'Informazione), University of Naples Federico II, Naples, Italy; ^4^ Institute of Robotics and Mechatronics, German Aerospace Center, Weling, Germany; ^5^ Department of Information Engineering and Centro E. Piaggio, University of Pisa, Pisa, Italy

**Keywords:** manipulation, end-effectors, biologically-inspired robots, robot control, robot perception

## 1 Editorial

In the past, robots have been almost exclusively designed with industrial settings in mind. Accurate positioning and precise tracking were the main requirements for performing repetitive tasks in structured environments with rigid end-effectors. In recent years, robots have come out of the cages Albu-Schaffer et al. (2008) and can freely interact and collaborate with humans in unstructured scenarios. This has opened new challenges to guarantee the safety of human-robot interaction and the purposeful interaction with the environment. To this aim, soft robots that embed compliant elements in their structure have emerged as a promising solution, and compliant end-effectors have been introduced, which can interact and adapt to external conditions as human hands do, have gained increasing attention.

This new framework, which encompasses the design, planning, sensing, and control of soft end-effectors, comes with several theoretical and practical open challenges. Indeed, the research community has made a considerable effort to develop novel academic and technological solutions to allow robots to operate amongst and with humans and safely move in, and interact with, unknown and unstructured environments.

Still, the question of how to use embedded compliance to enhance the interaction with the environment exploiting the multiple degrees of freedom of a soft manipulator is an open problem (Billard, 2019).

Indeed, a valid formalization could be to exploit model-based optimization approaches. An alternative approach is the usage of learning techniques to generalize from data acquired through tactile sensing. Another promising strategy is understanding how humans solve the problem at the muscular-skeletal level, with the ultimate goal to produce a limited yet effective replication of human-inspired solutions.

The manuscripts collected in this e-book shed some light on the usage of soft end effectors to interact with the surrounding world, presenting advanced design, control, and tactile sensing solutions.

## 2 Novel Design Ideas to Target Current Challenges in Soft Robotic Manipulation

Although several design solutions for soft end effectors have been proposed by the research community in the last decade, they are still far from the complexity and the capabilities of human hands, especially when the task is to grasp and manipulate in an unstructured and challenging environment. A typical example relies upon large warehouses, in which robots should quickly grasp and relocate a wide range of objects. In those cases, anthropomorphic robotic hands may fail in the case of high-speed tasks. A method to bridge this gap was proposed in Kakogawa et al. through the development of an under-actuated mechanism that can grasp and pull a wide range of different objects. The two actions are performed by one single actuator, producing a substantial complexity reduction w.r.t. conventional designs. The implementation can be performed in multiple ways, including two solutions presented by the authors: one driven by a DC motor with planetary gear reducers and the second driven by pneumatic actuators with branch tubes as a differential. In both cases, the pulling-in mechanism is achieved by a belt-driven finger surface and a linear slider with an air cylinder, respectively. Interestingly, such a design enables a combination of grasping and pulling actions, increasing the dexterity of classic under-actuated end-effectors.

However, the flexibility granted by soft hands can even go beyond the hand itself and be further expanded by the introduction of an additional degree of flexibility brought in by the possibility of quickly locking and unlocking the gripper from the robotic arm. This novel paradigm, introduced in Iqbal et al., presents the chance to exploit collaborative grippers that can be easily attached and detached from the robot arm and also used independently from it. The authors demonstrated with experiments involving 12 participants that this solution can increase the number of working conditions in which compliant robotic hands, complemented with tactile feedback to support the magnetic tool changer, can enhance human activity.

Of note, the anthropomorphic design is not per se the optimal solution in all the cases. On the contrary, following Occam’s Razor principle, non-anthropomorphic solutions should be preferred for certain tasks. This is the core of the interesting perspective discussion proposed in Hao and Visell, in which the Authors discuss how emerging soft robotic technologies applied to non-anthropomorphic grippers have yielded new techniques that can circumvent fundamental challenges associated with grasping via fingered grippers. Mechanisms for morphological deformation or adhesion, which simplify the grasping of diverse objects in different poses without detailed knowledge of the object geometry, are common to many non-anthropomorphic soft grippers are. These solutions could enable the usage of robotic manipulation in challenging applications, such as logistics or rapid manufacturing, with lower cost and complexity. Yet, many challenges, reviewed in Hao and Visell, remain open, and several research directions are ready to be explored.

## 3 Techniques to Exploit the Enhanced Capabilities of Soft End-Effectors

As briefly mentioned above, the avenue of innovative soft grippers design has offered several benefits, such as reduced cost, enhanced compliance, and customized design, with the promise of dexterity and robustness. However, to harness these characteristics, it is crucial to develop new tools to understand the capabilities of such manipulators and to facilitate manipulation planning with soft robotic grippers that exhibit free-form deformations. This topic is discussed in Sun et al. through the development of a sampling-based approach to discover and model continuous families of manipulations for soft robot hands. The method consists of sampling the space of manipulation actions, constructing Gaussian Mixture Model representations covering successful regions, and refining the results to create continuous successful regions representing the manipulation family. Authors considered models with and without dimensionality reduction, to deal with the high dimensionality of the manipulation actions space. This way, they provide a methodological approach to compare models defined with different dimensions. Interestingly, the Authors found that, albeit dimensionality reduction is typically helpful to populate the models, it is not easy to determine *a priori* the size of the low-dimensional domain because this may depend on the gripper and the task. These models can be used to plan and carry out successful and robust manipulation actions and compare competing robot hand designs.

## 4 Adaptable Tactile Sensing for Soft Robotic Hands

The studies of how robots should interact with the surrounding environment often focus on the actions applied to the external object. However, the dual problem of sensing the contacts is of equal importance. Indeed, the extraordinary manipulation capabilities of humans strongly depend and rely on the advanced tactile sensing system spread over our body and skin. In Hughes et al., the Authors proposed a compelling analysis on sensors morphology and structure that, for a given task, can significantly aid and improve tactile sensing capabilities. This can be done by relying on mechanisms such as improved sensitivity or morphological computation. Indeed, different tactile tasks can require different morphologies: this poses significant challenges related to the optimal design of sensors, which should ideally be variable. In Hughes et al., the Authors developed a jamming filter which, when placed over a tactile sensor, allows the filter to be shaped and molded online, thus varying the sensor structure. This solution demonstrated to be beneficial for sensory tasks since an adaptive structure can be used to alter and improve the amount and the quality of the information gathered through the sensors.
